# Collision Avoidance Path Planning and Tracking Control for Autonomous Vehicles Based on Model Predictive Control

**DOI:** 10.3390/s24165211

**Published:** 2024-08-12

**Authors:** Ding Dong, Hongtao Ye, Wenguang Luo, Jiayan Wen, Dan Huang

**Affiliations:** 1 School of Automation, Guangxi University of Science and Technology, Liuzhou 545036, China; 221068297@stdmail.gxust.edu.cn (D.D.); wgluo@gxust.edu.cn (W.L.); wenjiayan2012@126.com (J.W.); 2 Guangxi Key Laboratory of Automobile Components and Vehicle Technology, Guangxi University of Science and Technology, Liuzhou 545036, China; 3 School of Mechanical and Automotive Engineering, South China University of Technology, Guangzhou 510641, China; dan78huang@scut.edu.cn

**Keywords:** trajectory tracking, model predictive control, active collision avoidance, adaptive cruise control, path planning, alternating direction multiplier method

## Abstract

In response to the fact that autonomous vehicles cannot avoid obstacles by emergency braking alone, this paper proposes an active collision avoidance method for autonomous vehicles based on model predictive control (MPC). The method includes trajectory tracking, adaptive cruise control (ACC), and active obstacle avoidance under high vehicle speed. Firstly, an MPC-based trajectory tracking controller is designed based on the vehicle dynamics model. Then, the MPC was combined with ACC to design the control strategies for vehicle braking to avoid collisions. Additionally, active steering for collision avoidance was developed based on the safety distance model. Finally, considering the distance between the vehicle and the obstacle and the relative speed, an obstacle avoidance function is constructed. A path planning controller based on nonlinear model predictive control (NMPC) is designed. In addition, the alternating direction multiplier method (ADMM) is used to accelerate the solution process and further ensure the safety of the obstacle avoidance process. The proposed algorithm is tested on the Simulink and CarSim co-simulation platform in both static and dynamic obstacle scenarios. Results show that the method effectively achieves collision avoidance through braking. It also demonstrates good stability and robustness in steering to avoid collisions at high speeds. The experiments confirm that the vehicle can return to the desired path after avoiding obstacles, verifying the effectiveness of the algorithm.

## 1. Introduction

With the development of automotive science and technology, the importance of autonomous vehicles is becoming increasingly prominent. Their practicality in both military and commercial applications continues to grow [1]. Obstacle avoidance is a critical capability of autonomous vehicles. It refers to the ability to perceive the vehicle’s surroundings through sensors and control the vehicle to safely navigate around obstacles without collisions. In vehicular safety incidents, rear-end collisions account for a significant proportion [2]. Rear-end collisions often result from inadequate following distance, driver distraction, and unexpected sudden traffic congestion [3]. Currently, collision avoidance systems are becoming standard auxiliary systems for autonomous vehicles. Depending on specific traffic situations, they are categorized into two collision avoidance methods: steering and braking [4].

The rapid development of vehicle sensing technology has enabled ACC to effectively reduce the occurrence of rear-end collisions. However, in dynamic urban road environments, active steering collision avoidance is often required to ensure the safety of vehicle operation [5,6,7,8]. Since then, many different obstacle avoidance methods for autonomous vehicles and various successful implementations have been reported in the literature. These methods aim to enable vehicles to navigate swiftly and steadily through obstacles. Among numerous obstacle avoidance algorithms, MPC is the most widely used technique [9]. Using the MPC framework can improve the tracking accuracy of ACC [10]. Combining hybrid system theory with multi-objective ACC, in multi-target traffic scenarios, the objective function is modeled as a quadratic cost function of discrete-time piecewise affine systems. The optimal state feedback control law was found by solving the finite-time optimal control problem with dynamic programming [11]. An ACC system model is established using self-aware vehicles and inter-vehicle dynamics. Based on an MPC method utilizing Laguerre orthogonal basis functions, the complexity of the optimization problem in ACC applications is reduced [12]. Combine MPC with ACC to design a variable-weight model predictive controller. It employs the gaussian naive bayes algorithm to predict the future behavior of vehicles ahead and dynamically adjust the weighting coefficients in real-time [13]. All the above-mentioned references focus solely on longitudinal dynamics and do not consider steering maneuvers for obstacle avoidance.

If braking control alone cannot achieve collision avoidance, active steering must be employed to avoid collisions with obstacles. Typically, a hierarchical two-level controller is employed, comprising an upper-level path planning controller and a lower-level trajectory tracking controller. To improve the vehicle’s obstacle avoidance capability, an obstacle avoidance control strategy based on the vehicle’s lateral dynamics model is proposed. This strategy includes a forbidden zone penalty function and prediction distance multiplication. Its effectiveness is validated through simulation experiments. However, it does not consider dynamic obstacles [14]. The motion planner, based on NMPC, enhances computation speed by reducing optimization variables. It simplifies non-convex constraints, ensuring driving safety. Additionally, it achieves a good balance between computational load and accuracy. However, the motion planning performance is poor in dynamic environments [15]. Using adaptive segmented Bezier curves, dynamic environment obstacle avoidance trajectory planning is conducted for autonomous vehicles. The stability of the vehicle during high-speed obstacle avoidance is verified through simulation. However, emergency braking of the vehicle was not considered during the avoidance process [16]. Combining event triggering with MPC, motion planning and control for autonomous vehicles are conducted. This is based on trajectory tracking errors as triggering conditions at different frequencies. It reduces computational costs while ensuring driving safety. However, the article only considers static obstacles [17]. In motion planning considering predictive risk, predictions of future trajectories of surrounding vehicles are integrated. This helps assess driving risks. When surrounding vehicles engage in emergency acceleration or deceleration, risks can be effectively avoided, thus enhancing driving safety. However, the considered vehicle model is relatively simple, and high-speed scenarios are not taken into account [18]. Using MPC, a hierarchical structured path planning and tracking controller is developed. This enables the planning of safe avoidance paths, allowing vehicles to respond to emergency situations. However, the considered vehicle speed is relatively low [19]. Based on the improved potential field model and hidden markov model, trajectory tracking is conducted using MPC. This enables dynamic obstacle avoidance in complex traffic environments. However, driver characteristics and emergency conditions are not considered in the paper [20]. Combining convex approximation principles of obstacle avoidance with MPC, comprehensively considering factors such as obstacle vehicle shapes, road constraints, road centering, and priority for left lane changing to achieve path planning in complex dynamic traffic environments. However, the detailed decision-making process complicates the control mode, which may hinder system stability [21]. Combining the adaptive speed zone controller and the path planning controller enhances speed adaptability. It also generates optimal paths through multiple obstacles. However, the impact of preceding vehicle acceleration changes on the system is not considered [22]. A secure and anti-collision multi-platoon control design approach is proposed to ensure the desired inter-platoon and intra-platoon tracking performance with a collision-free guarantee. It is formally proved that the inter- and intra-platoon tracking errors converge to small neighborhood around zero [23].A bandwidth parameter-dependent co-design approach is proposed for each train to determine the desired cruise controller gains and event scheduler parameters. Furthermore, under the designed cruise controllers, the follower trains can maintain the desired gap reference from their predecessors and keep the same speed profile with the leader train, while simultaneously realizing promising communication resource efficiency [24].

This paper addresses the situation where autonomous vehicles cannot meet obstacle avoidance requirements using emergency braking alone. Building upon ACC, the paper introduces active steering control for autonomous vehicles. A hierarchical controller is adopted, where the upper-level planner employs NMPC for path planning based on the vehicle’s kinematic model. It designs obstacle avoidance functions based on the relative distance between the ego vehicle and obstacle vehicles, which is obtained through onboard LiDAR detection in real-time. This distance, combined with a safety distance model, informs the decision-making model for unmanned driving vehicle braking/steering while maintaining vehicle following. The lower controller, based on the vehicle’s three degrees of freedom model, utilizes MPC for trajectory tracking. Finally, a CarSim/Simulink integrated simulation platform is constructed to verify the proposed algorithm under three scenarios: high-speed avoidance of dynamic obstacles in dual-line conditions, avoidance of static obstacles in straight-line conditions, and following scenarios in straight-line conditions.

The organization of this article is as follows. The overall control framework for vehicle braking and steering is introduced in Section 2. The combination of the upper controller MPC with ACC and the design of the lower controller are presented in Section 3. The design of the switching strategy and improvements to the obstacle avoidance function are shown in Section 4. Section 5 validates the proposed controller through three scenarios, and comparing the real-time performance of the controller improved with ADMM to that of the traditional controller during obstacle avoidance. Finally, a brief conclusion is given in Section 6.

## 2. System Control Framework

**Figure 1:**
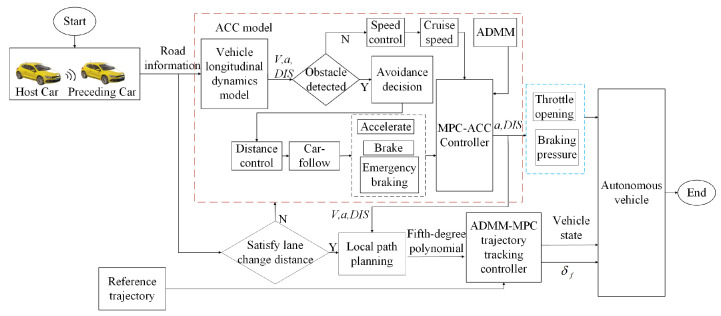
The kinematic relationship between the two vehicles.

The overall system control framework described in the paper utilizes a hierarchical controller approach. The upper layer uses NMPC for path planning, focusing on obstacle avoidance by analyzing real-time data from onboard LiDAR. The lower layer employs MPC for trajectory tracking of the planned path, as shown in Figure 1. Host car is the controlled vehicle, commonly referred to as the ego vehicle. Preceding car is the lead vehicle whose behavior (speed, acceleration, etc.) is used as a reference for the host car to follow. The upper layer uses NMPC to perform path planning based on the kinematic model, and designs the obstacle avoidance function based on the relative distance between the autonomous vehicle and obstacles. This relative distance is detected in real time by the vehicle-mounted LiDAR and combined with the safe distance model to design a decision-making model for braking/steering when following the vehicle. In order to solve the problem that autonomous vehicles cannot avoid obstacles by emergency braking alone, active steering control based on adaptive cruise control is introduced. The MPC-ACC ensures that the host vehicle and the preceding vehicle maintain a safe distance while optimizing the speed based on a series of conditions such as the relative distance and speed of the two vehicles. We apply ADMM in the rolling optimization of MPC-ACC to enhance the controller’s solving speed. The lower controller uses the improved MPC trajectory tracking controller to track the local planned path. Finally, a CarSim/Simulink co-simulation platform is built to validate the proposed algorithm in three scenarios: high-speed obstacle avoidance under double lane change conditions, obstacle avoidance under straight-line conditions, and following a vehicle under straight-line conditions. The real-time performance of the improved controller is compared with that of the unimproved controller in these scenarios.

## 3. Model Establishment

In this section, we describe the hierarchical control framework utilized in our autonomous driving system, which consists of two main controllers, the upper controller and the lower controller. Each controller has distinct roles and functions, which are crucial for the overall system’s performance.

The upper controller utilizes NMPC for path planning, dynamically avoiding obstacles using real-time data from onboard LiDAR sensors, optimizing the path based on current road conditions and the surrounding environment, and making high-level strategic decisions to ensure safety and efficiency. The lower controller employs MPC for trajectory tracking, executing the path provided by the upper controller with real-time adjustments to the vehicle’s trajectory. It calculates the necessary control inputs, such as steering angles, acceleration, braking, and handles ACC to maintain a safe distance from the preceding vehicle by solving a quadratic programming problem using the ADMM for precise speed adjustments.

### 3.1. Upper Controller Design

#### 3.1.1. Car-Following Mode

The car-following mode is the main function of ACC, and it directly influences the performance of the entire ACC controller. Therefore, this section develops an ACC controller based on MPC [25].

**Figure 2:**
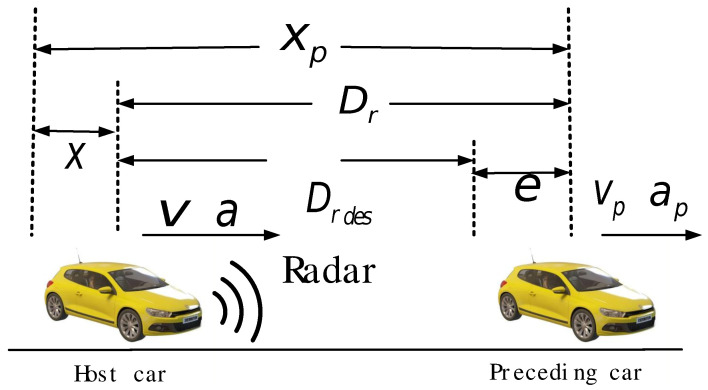
The kinematic relationship between the two vehicles.

By analyzing the kinematic relationship between the two vehicles, as shown in Figure 2, and considering the acceleration and acceleration rate of the two vehicles, the kinematic equations of the two vehicles are obtained as follows:

(1) $$\left\{\begin{array}{c} D_{r}\left(k+1\right)=D_{r}\left(k\right)+v_{rel}\left(k\right)T+\frac{T^{2}}{2}\left(a_{p}\left(k\right)-a\left(k\right)\right)\\ v(k+1)=v(k)+a(k)T\\ v_{rel}\left(k+1\right)=v_{rel}\left(k\right)+\left(a_{p}\left(k\right)-a\left(k\right)\right)T\\ a\left(k+1\right)=\left(1-\frac{T}{\tau }\right)a\left(k\right)+\frac{T}{\tau }a_{des}\\ j\left(k+1\right)=-\frac{1}{\tau }a\left(k\right)+\frac{1}{\tau }a_{des} \end{array}\right.$$

where $D_{r}\left(k\right)$ is the relative displacement, $v_{rel}\left(k\right)$ is the relative velocity, v(k) is the velocity of the host vehicle, $a_{p}\left(k\right)$ is the acceleration of the lead vehicle, a(k) is the acceleration of the host vehicle, *T* is the time constant of the lower controller, $a_{des}$ represents the desired acceleration, and j(k) denotes the rate of change of acceleration.

Taking $x\left(k\right)={\left[D_{r}\left(k\right),v\left(k\right),v_{rel}\left(k\right),a\left(k\right),j\left(k\right)\right]}^{T}$ as the state variable and $u=a_{des}$ as the control variable, the above equations are transformed into state-space representation as follows:

(2) $$x\left(k+1\right)=Ax\left(k\right)+B_{1}u_{1}\left(k\right)+B_{2}u_{2}\left(k\right)$$

where $A=\left[\begin{array}{ccccc} 1 & 0 & T & -\frac{T}{2} & 0\\ 0 & 1 & 0 & T & 0\\ 0 & 0 & 1 & -T & 0\\ 0 & 0 & 0 & 1-\frac{T}{\tau } & 1\\ 0 & 0 & 1 & -\frac{1}{\tau } & 0 \end{array}\right]$, $B_{1}={\left[\begin{array}{ccccc} 0 & 0 & 0 & \frac{T}{\tau } & \frac{1}{\tau } \end{array}\right]}^{T}$, $B_{2}={\left[\begin{array}{ccccc} \frac{T^{2}}{2} & 0 & T & 0 & 0 \end{array}\right]}^{T}$, $u_{1}=a_{des}$, $u_{2}=a_{p}$.

Selecting $y\left(k\right)={\left[e\left(k\right),v_{rel}\left(k\right),a\left(k\right),j\left(k\right)\right]}^{T}$ as the output variable of the system, where e(k) represents the distance error, the state equation of the system can be expressed as follows:

(3) $$\left\{\begin{array}{c} x\left(k+1\right)=Ax\left(k\right)+B_{1}u_{1}\left(k\right)+B_{2}u_{2}\left(k\right)\\ y(k)=Cx(k)-Z \end{array}\right.$$

where *C* is the output matrix, and $d_{0}$ represents the minimum safety distance. $C=\left[\begin{array}{ccccc} 1 & 0 & 0 & 0 & 0\\ 0 & 0 & 1 & 0 & 0\\ 0 & 0 & 0 & 1 & 0\\ 0 & 0 & 0 & 0 & 1 \end{array}\right]$, $Z={\left[\begin{array}{cccc} d_{0} & 0 & 0 & 0 \end{array}\right]}^{T}$, and in this paper, $d_{0}$ is set to 5 m.

#### 3.1.2. Predictive Model and Objective Function Design

The future behavior of the ACC system is forecasted using MPC, which generates prediction equations within the specified range of the above equation.

(4) $$\left\{\begin{array}{c} {\tilde{X}}_{p}\left(k+N_{p}|k\right)=\bar{A}x\left(k\right)+{\bar{B}}_{1}U_{1}\left(k+N_{c}\right)+{\bar{B}}_{2}U_{2}\left(k+N_{p}\right)+\bar{G}e\left(k\right)\\ {\tilde{Y}}_{p}\left(k+N_{p}|k\right)=\bar{C}x\left(k\right)+\bar{D}U_{1}\left(k+N_{c}\right)+\bar{E}U_{2}\left(k+N_{p}\right)+\bar{F}e\left(k\right)-\bar{Z} \end{array}\right.$$

where ${\tilde{X}}_{p}\left(k+N_{p}|k\right)$ represents the set of predicted state variables, ${\tilde{Y}}_{p}\left(k+N_{p}|k\right)$ represents the set of predicted outputs, U(k+n) represents the output variable sequence. $\bar{A}$ and $\bar{C}$ represent system matrices, ${\bar{B}}_{1}$ and $\bar{D}$ represent input matrices, ${\bar{B}}_{2}$ and $\bar{E}$ represent disturbance matrices, $\bar{G}$ and $\bar{F}$ represent error matrices, and $\bar{Z}={\left[\begin{array}{cccc} Z & Z & \cdots & Z \end{array}\right]}^{T}$.

Using the MPC framework, design the following objective function. The control objective is to ensure that the vehicle can accurately track the desired speed and acceleration, while also making the control process smoother.

(5) $$J=\sum_{j=1}^{N_{p}}{\left[{\tilde{Y}}_{p}\left(k+j|k\right)-Y_{r}\left(k+j\right)\right]}^{T}Q\left[{\tilde{Y}}_{p}\left(k+j|k\right)-Y_{r}\left(k+j\right)\right]+\sum_{j=0}^{N_{c}-1}u{\left(k+j\right)}^{T}Ru\left(k+j\right)$$

where *Q* and *R* are the weight matrix of outputs and control inputs. The elements of *Q* is chosen based on the desired performance criteria, such as maintaining a safe distance from the lead vehicle and achieving smooth acceleration and deceleration. *R* is chosen to balance the need for precise control with the desire to minimize control effort, and *u* is the control vector matrix.

Predict the state x(k+j|k) of $N_{p}$ steps in the future based on the system model. Convert the objective function and constraints into an optimization problem, and generate a control input sequence by solving the optimization problem $\left\{u\left(k\right),u\left(k+1\right),\ldots ,u\left(k+N_{p}-1\right)\right\}$. The first control input in the sequence of inputs u(k) is applied to real systems.

Taking into account factors such as vehicle performance, velocity, acceleration, and desired acceleration, the constraint conditions are set as follows:

(6) $$\left\{\begin{array}{c} v_{\min}\leq v\left(k\right)\leq v_{\max}\\ a_{\min}\leq a\left(k\right)\leq a_{\max}\\ j_{\min}\leq j\left(k\right)\leq j_{\max}\\ u_{\min}\leq u\left(k\right)\leq u_{\max}\\ D_{r}\geq d_{0} \end{array}\right.$$

#### 3.1.3. Transformation and Solution of the Quadratic Programming Problem

Equation (5) establishes the general form of the objective function. To facilitate machine solving and obtain the optimal control sequence, it must be transformed into a quadratic programming problem. The constraints in Equation (6) can be organized as follows:

(7) MU(k+n)≤W

where $M=\left[\begin{array}{c} \bar{L}{\bar{B}}_{1}\\ -\bar{L}{\bar{B}}_{1}\\ I\\ I \end{array}\right]$, $W=\left[\begin{array}{c} \bar{N}-\bar{L}{\bar{B}}_{2}U_{2}\left(k+N_{p}\right)-\bar{L}\bar{A}x\left(k\right)-\bar{L}\bar{G}e\left(k\right)\\ -\bar{T}+\bar{L}{\bar{B}}_{2}U_{2}\left(k+N_{p}\right)+\bar{L}\bar{A}x\left(k\right)+\bar{L}\bar{G}e\left(k\right)\\ U_{\max}\\ -U_{\min} \end{array}\right]$, $N=\left[\begin{array}{c} Inf\\ v_{\max}\\ a_{\max}\\ j_{\max} \end{array}\right]$, $T=\left[\begin{array}{c} d_{0}\\ v_{\min}\\ a_{\min}\\ j_{\min} \end{array}\right]$, $L=\left[\begin{array}{ccccc} 1 & 0 & 0 & 0 & 0\\ 0 & 1 & 0 & 0 & 0\\ 0 & 0 & 0 & 1 & 0\\ 0 & 0 & 0 & 0 & 1 \end{array}\right]$, $\bar{T}=\left[\begin{array}{c} T\\ T\\ \vdots \\ T \end{array}\right]$, $\bar{L}=\left[\begin{array}{cccc} L & & & \\ & L & & \\ & & \ddots & \\ & & & L \end{array}\right]$.

At this point, Equation (5) can be transformed into the standard form of a quadratic programming problem, abbreviated as:

(8) $$\begin{array}{c} \min_{U}\frac{1}{2}U^{T}HU+f^{T}U\\ s.t.MU(k+n)\leq W \end{array}$$

The standard quadratic programming problem can be solved using MATLAB’s quadprog function. Based on our previous research on improving the MPC solver with ADMM [26]. The ADMM is a powerful optimization technique used to solve complex problems efficiently by breaking them down into simpler sub-problems. This section provides a detailed explanation of how ADMM enhances the performance of the solution process, especially in the context of the proposed MPC-ACC integration, and compares it with other optimization methods. ADMM combines the benefits of dual decomposition and augmented Lagrangian methods. It is particularly effective for optimization problems that can be decomposed into smaller sub-problems. ADMM can handle various constraints and objective functions, making it versatile for different types of optimization problems. The longitudinal speed control problem is formulated as a quadratic programming problem. ADMM efficiently decomposes and solves this problem, reducing computational load and improving real-time applicability. Interior point methods are powerful for solving convex optimization problems but can be computationally expensive for large-scale problems due to the need to solve large linear systems at each iteration. We apply ADMM in the rolling optimization of MPC to enhance the controller’s solving speed. Furthermore, the controller used for vehicle trajectory tracking in this paper is the MPC based on our proposed ADMM.

At each sampling instant, the vehicle detects the current environmental information through sensors and transfers its state variables to the MPC. Then, the above objective function is transformed into a quadratic programming problem for solution, obtaining the corresponding control sequence. The first value is applied to the control system, and the process is repeated at the next sampling instant.

### 3.2. Lower Controller Design

Equation (3) provides the state equation for the upper-level controller, forming the basis for controlling the vehicle’s longitudinal motion. However, since the output variables contain only velocity, acceleration, and relative distance, they cannot directly influence an autonomous vehicle. Therefore, a lower-level controller is needed to precisely control the vehicle’s braking and driving modes based on the desired acceleration. Converting the upper controller’s acceleration commands into brake pressure and throttle opening allows direct control over the vehicle’s motion [27].

This paper only considers air resistance and rolling resistance, assuming the vehicle travels on a flat road surface and neglecting road gradient resistance. Hence, the acceleration equation for the vehicle’s longitudinal motion [28] can be given by the following equation:

(9) $$ma_{tr}=F_{tr}=F_{aero}+F_{roll}$$

where *m* represents the vehicle mass, $a_{tr}$ represents the resistance acceleration, $F_{tr}$ represents the total resistance force, $F_{aero}$ represents the air resistance, and $F_{roll}$ represents the rolling resistance.

Rolling resistance $F_{roll}$ can be obtained from the following equation:

(10) $$F_{roll}=C_{R}mg$$

where $C_{R}$ represents the rolling resistance coefficient, and *g* represents the gravitational acceleration, here it is assumed that *g* is approximately 9.8 m/$s^{2}$.

The calculation method for air resistance can be obtained from the following equation:

(11) $$F_{aero}=\frac{1}{2}C_{E}S_{e}\kappa v^{2}$$

where $C_{E}$ represents the drag coefficient, $S_{e}$ represents the frontal area, and κ represents the air density, typically around 1.29 kg/$m^{3}$ for dry air.

During braking and driving mode transitions, it’s crucial to prevent simultaneous operation and avoid overly frequent switches, as this could affect vehicle comfort. The switching logic based on desired acceleration is as follows:

(12) $$\alpha_{thdes}=\left\{\begin{array}{c} \alpha_{thdes},a_{des}\geq -a_{tr}\\ 0,a_{des}<-a_{tr} \end{array}\right.$$

where $\alpha_{thdes}$ represents the desired throttle opening.

(13) $$P_{bdes}=\left\{\begin{array}{c} 0,a_{des}<-a_{tr}\\ P_{bdes},a_{des}\geq -a_{tr} \end{array}\right.$$

where $P_{bdes}$ represents the desired brake master cylinder pressure.

When the desired acceleration is greater than or equal to the required acceleration for overcoming resistance, the vehicle operates in driving mode. At this point, the driving force $F_{q}$ of the vehicle is:

(14) $$F_{q}=\rho ma_{des}+F_{roll}+F_{aero}$$

where the conversion factor for rotational mass ρ can be obtained from an empirical formula.

At this point, the required output torque of the engine can be calculated from the following equation:

(15) $$T=\frac{F_{e}R_{e}}{i_{g}i_{0}\eta }$$

where $R_{e}$ represents the radius of the wheels, $i_{g}$ represents the transmission gear ratio, $i_{0}$ represents the differential gear ratio, and η represents the mechanical efficiency of the transmission system.

The throttle opening in driving mode can be obtained through the relationship between engine speed, throttle opening, and engine output torque.

(16) $$\alpha_{thdes}={T_{MAP}}^{-1}\left(N,T\right)$$

where *N* represents engine speed, and $T_{MAP}$ represents the relationship expression among them.

When the vehicle operates in braking mode, the vehicle’s braking demand torque can be obtained from the following equation:

(17) $$F_{b}=-ma_{des}-F_{roll}-F_{aero}$$

The brake master cylinder pressure can be obtained from the following equation:

(18) $$P_{bdes}=\frac{F_{b}}{k_{b}}$$

where $k_{b}$ represents the ratio of braking force to brake master cylinder pressure.

Equations (16) and (18) provide the desired throttle opening and brake master cylinder pressure for the vehicle in acceleration and braking modes, respectively. These values directly control the vehicle to maintain the desired speed.

## 4. The Combination of Adaptive Cruise Control and Obstacle Avoidance

### 4.1. Designing Mode Switching Conditions

This paper integrates the vehicle’s adaptive cruise control with autonomous obstacle avoidance capabilities, dividing the driving phase into three different modes: following mode, braking mode, and emergency steering mode. Here, the relative distance to the vehicle ahead is used as the mode switching condition. The radar sensor detects different relative distances to control the vehicle’s transition to the corresponding driving mode.

#### 4.1.1. Car-Following Distance

The vehicle normally travels at a constant speed of 120 km/h. When an obstacle appears in front, the desired following distance is obtained from the safety distance model. When the following distance is met, the vehicle enters the following mode. This system adopts the variable time headway (VTH) [29] strategy, whose expression is as follows:

(19) $$S_{x}=v_{f}t_{h}+d_{0}$$

where $S_{x}$ represents the desired following distance, $t_{h}$ represents the headway time, $v_{f}$ represents for the vehicle speed, and $d_{0}$ represents the minimum safe distance, typically including the minimum distance between vehicles and the vehicle’s length. The calculation method for $t_{h}$ is as follows:

(20) $$t_{h}=t_{0}-c_{a}v_{rel}$$

where $t_{0}$ and $c_{a}$ are both positive constants, and $v_{rel}$ represents the relative velocity between the host vehicle and the obstacle vehicle.

#### 4.1.2. Braking Distance

When the vehicle is emergency braking or driving at very low speeds and satisfies the switching conditions, it applies brake to avoid collision. To simplify calculations, the vehicle is set to brake with maximum braking cylinder pressure, and a safety distance model is established based on the braking process to calculate the braking distance. The expression for the longitudinal safety distance during the braking process is:

(21) $$S_{z}=S_{f}+d_{0}-S_{d}$$

where $S_{f}$ represents the braking displacement of the host vehicle during the braking process, while $S_{d}$ represents the braking displacement of the preceding vehicle during the braking process.

The braking displacement of the host vehicle is:

(22) $$S_{f}=v_{f}t+\frac{1}{6}J_{\max}t^{3}+\frac{1}{2}a_{\max}{t_{1}}^{2}$$

where *t* represents the time required for acceleration from zero to maximum value, $t_{1}$ denotes the time taken for the vehicle speed to decrease from the current speed to zero, and $J_{max}$ represents the deceleration gradient.

#### 4.1.3. Emergency Steering Distance

When longitudinal emergency braking cannot prevent a collision, the vehicle needs to actively steer to avoid obstacles. At this point, the vehicle enters the emergency steering mode. The steering safety distance $S_{c}$ represents the longitudinal displacement from the host vehicle to the preceding vehicle at the critical collision moment, it can be expressed as:

(23) $$S_{c}=v_{rel}\left(t_{1}-t_{0}\right)+\int_{0}^{t_{1}}\int_{0}^{\sigma }a_{rel}dtd\delta$$

where $v_{rel}$ represents the relative velocity between the host vehicle and the obstacle vehicle, $a_{rel}$ represents the relative acceleration, while $t_{0}$ and $t_{1}$ represent the start time of the turn and the collision time. Due to the short duration of the collision process, it is assumed here that the speed of the host vehicle remains unchanged throughout the process, thus the equation can be simplified to:

(24) $$S_{c}=v_{rel}\left(t_{1}-t_{0}\right)$$

### 4.2. Obstacle Avoidance Planning

#### 4.2.1. Obstacle Avoidance Function

The information about obstacles can be obtained through radar scans, and the penalty function is generally implemented using an inverse proportion function of the distance between the host vehicle and the obstacle, as shown in the following equation:

(25) $$J_{obs}=\frac{Sv}{{\left(x_{0}-x_{obs}\right)}^{2}+{\left(y_{0}-y_{obs}\right)}^{2}+\varepsilon }$$

where $\left(x_{0},y_{0}\right)$ represents the current position of the vehicle’s center of mass, $\left(x_{obs},y_{obs}\right)$ represent the coordinates of the obstacle, *S* represents the obstacle avoidance weighting coefficient, ε>0 is to prevent the denominator from being zero, and *v* represents the current velocity of the vehicle.

Although this method can avoid obstacles, it does not consider the vehicle’s dimensions and cannot accurately delineate obstacle boundaries. Errors may occur in the obstacle avoidance prediction process, such as erroneous results suggesting the vehicle passing through obstacles. Even if a path avoiding obstacles can be planned, collisions may occur due to proximity to obstacles. Therefore, this paper improves the obstacle avoidance function by incorporating the emergency steering distance obtained in the previous section. Considering the critical collision distance, the improved penalty function ensures that the planned path does not bring the vehicle too close to obstacles, thus avoiding collisions. The obstacle avoidance function is expressed as follows:

(26) $$J_{obs}=\left\{\begin{array}{c} 0,DIS>S_{c}\\ M,DIS\leq S_{c} \end{array}\right.$$

where *M* represents a sufficiently large positive number, and DIS represents the real-time relative distance between the ego vehicle and the obstacle, which can be detected by radar. When the relative distance satisfies the conditions for emergency steering, the presence of *M* causes a sharp increase in the function value of the path planner, forcing the path planner to re-search for the optimal path.

Due to the large computational complexity of trajectory replanning, using the vehicle dynamics model may not allow for real-time planning of avoidance paths, leading to collisions. To simplify the computation, the planning layer adopts a kinematic model. Setting the state variable $\xi ={\left[\dot{y},\dot{x},\phi ,Y,X\right]}^{T}$, the discretized prediction of the state variable k+1 at the next time step *k* under the current time step k+1 is:

(27) $$\begin{array}{c} \xi \left(k+1\right)={\left[\begin{array}{ccccc} y(k) & x(k) & \phi (k) & Y(k) & X(k) \end{array}\right]}^{T}=\\ \left[\begin{array}{c} v\left(k\right)\sin\delta_{f}\\ v\left(k\right)\cos\delta_{f}\\ \phi (k)\\ Y(k)+T[x(k)\sin\phi (k)+y(k)\cos\phi (k)]\\ X(k)+T[x(k)\cos\phi (k)-y(k)\sin\phi (k)] \end{array}\right] \end{array}$$

where *v* represents the vehicle velocity, $\delta_{f}$ represents the front wheel steering angle, and ϕ represents the vehicle’s yaw angle. Selecting $\delta_{f}$ as the control variable for trajectory replanning, the control objective of trajectory replanning is to minimize the deviation between the actual trajectory and the reference trajectory while avoiding obstacles. The objective function of the trajectory planning layer can be designed as:

(28) $$\left\{\begin{array}{c} J=\min_{U}\sum_{i=1}^{N_{p}}||\eta \left(k+i|k\right)-\eta_{r}{\left(k+i|k\right)||}_{Q}^{2}+\left|\right|U_{i}{\left|\right|}_{R}^{2}+J_{obs}\\ J_{obs}=\left\{\begin{array}{c} 0,DIS>S_{c}\\ M,DIS\leq S_{c} \end{array}\right.\\ s.t.U_{\min}\leq U\leq U_{\max} \end{array}\right.$$

where $\eta_{r}$ represents the reference trajectory, and $U=(\delta_{f})$ represents the front wheel steering angle.

The planning layer has relatively low real-time performance. Using a higher-precision NMPC can fully meet the requirements of trajectory replanning. Therefore, the above objective function is no longer linearized, but solved using a nonlinear model. By solving it, the optimal front wheel steering angle for the vehicle at the current position is obtained. Because MPC has a feedback correction loop, the control variable is incorporated into Equation (26) to update the values of various state variables of the vehicle, and then rolling optimization is performed in the next cycle.

#### 4.2.2. Trajectory Fitting

The trajectory replanning results in discrete points within the prediction horizon. Directly inputting these points into the interface of the control layer would consume a large amount of resources and cannot be directly fed into the control layer. Therefore, it is necessary to process the local reference path obtained from the replanning. Curve fitting is the main method for handling discrete points. Considering the first and second-order continuous dynamic characteristics required by the vehicle model, this paper considers using the quintic polynomial as the fitting curve, expressed as follows:

(29) $$\left\{\begin{array}{c} Y=a_{0}t^{5}+a_{1}t^{4}+a_{2}t^{3}+a_{3}t^{2}+a_{4}t+a_{5}\\ \phi =b_{0}t^{5}+b_{1}t^{4}+b_{2}t^{3}+b_{3}t^{2}+b_{4}t+b_{5} \end{array}\right.$$

Parameter vector $\alpha =\left[\begin{array}{cccccc} a_{0} & a_{1} & a_{2} & a_{3} & a_{4} & a_{5} \end{array}\right]$ from the lateral displacement polynomial and parameter vector $\beta =\left[\begin{array}{cccccc} b_{0} & b_{1} & b_{2} & b_{3} & b_{4} & b_{5} \end{array}\right]$ from the vehicle yaw angle polynomial are input to the control layer.

### 4.3. Overall Mode Switching Strategy Design

**Figure 3:**
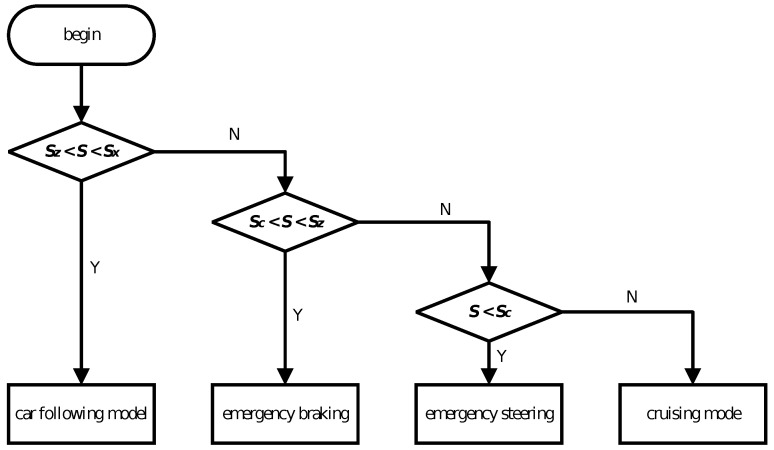
Mode switching control block diagram.

Based on the first two sections, the control logic for the overall mode switching is designed. The control logic is illustrated in the figure below:

In Figure 3, $S_{c}$, $S_{z}$, and $S_{x}$ represent various mode switching conditions, where $S_{x}$ represents the following distance, $S_{z}$ represents emergency braking distance, and $S_{c}$ represents emergency steering distance. When the conditions are met, the system enters the corresponding mode; otherwise, it continues to maintain the current mode.

(1) When the relative distance is between the emergency braking distance and the following distance, the vehicle enters the following mode.

(2) When the relative distance is between the emergency steering distance and the emergency braking distance, the vehicle enters the emergency braking mode.

(3) When the relative velocity reaches the emergency steering distance, the vehicle enters the emergency steering mode. After completing the steering maneuver, the vehicle returns to the reference path and continues stable driving at cruise speed or the speed of the vehicle ahead.

## 5. Simulation Validation

To validate the effectiveness of the proposed algorithm, a joint simulation platform using Simulink and CarSim is established. The vehicle parameters used in the simulation are shown in Table 1.

The simulation experiments consist of three test scenarios: high-speed avoidance of moving obstacles in a dual-lane situation, braking and steering to avoid stationary obstacles, and following another vehicle. In CarSim, the lead vehicle is set to be driver-operated and applies to all scenarios.

### 5.1. The Scenario of Dynamic Obstacle Avoidance in a Dual-Lane Situation

**Figure 4:**
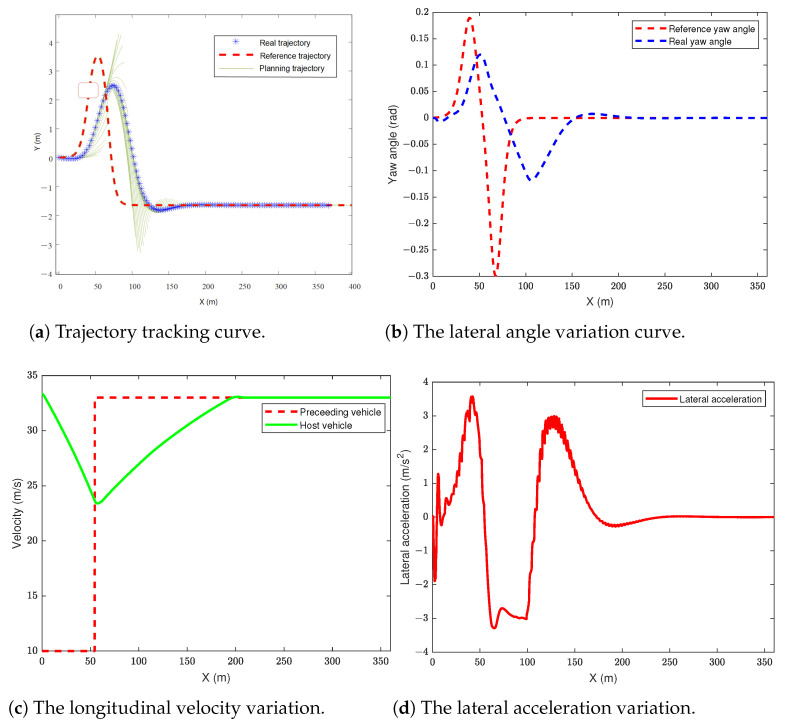
Scenarios for Adaptive Cruise Control.

In this section of the simulation, the dual-lane scenario is used as the reference trajectory. The autonomous vehicle travels at 120 km/m, while the obstacle vehicle travels at 36 km/m in the left lane of the autonomous vehicle. The simulation results are shown in Figure 4.

From Figure 4a, it can be observed that the preceding vehicle maintains a constant speed at approximately 35 m to the left front of the host vehicle, passing the reference trajectory of the host vehicle at a longitudinal distance of 50 m, as indicated by the red box in the figure. The vehicle performs local path planning during trajectory tracking, successfully avoiding the moving obstacle to the left front while being able to return to the reference trajectory. Figure 4b depicts the change in the vehicle’s yaw angle. Due to the newly planned trajectory during obstacle avoidance, the actual yaw angle variation is smaller than the reference yaw angle variation, ensuring vehicle stability. Additionally, after completing the obstacle avoidance, the actual yaw angle smoothly tracks the reference yaw angle. Figure 4c illustrates the variation in the host vehicle’s longitudinal velocity. The speed of the preceding vehicle detected by the onboard sensor serves as the reference velocity for the host vehicle. During obstacle avoidance, the host vehicle decelerates and brakes, and after clearing the obstacle to the left front, it detects no obstacles ahead and resumes cruising speed. Figure 4d shows the lateral acceleration of the vehicle. It can be observed that the lateral acceleration during steering collision avoidance remains within 3.7 m/$s^{2}$, not exceeding 0.4 g, indicating that the tires operate within the linear region during obstacle avoidance, maintaining vehicle stability throughout the process.

### 5.2. Emergency Braking and Steering Avoidance Scenario

**Figure 5:**
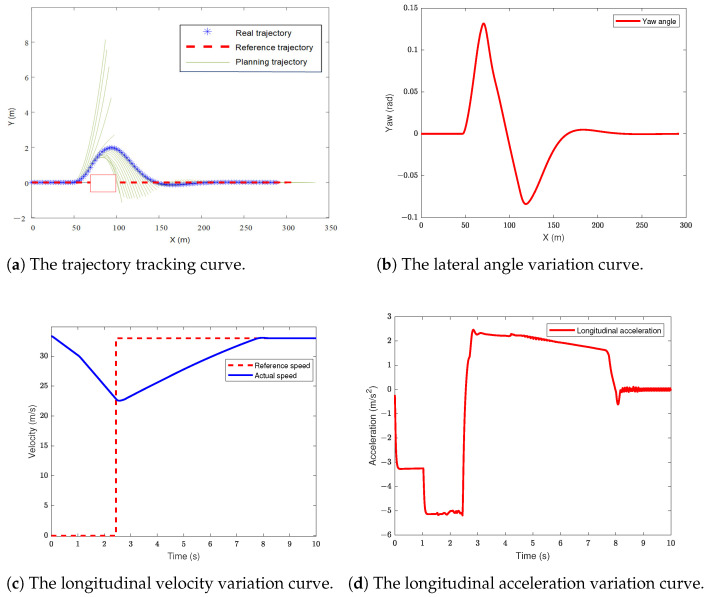
Scenarios for Adaptive Cruise Control.

**Figure 6:**
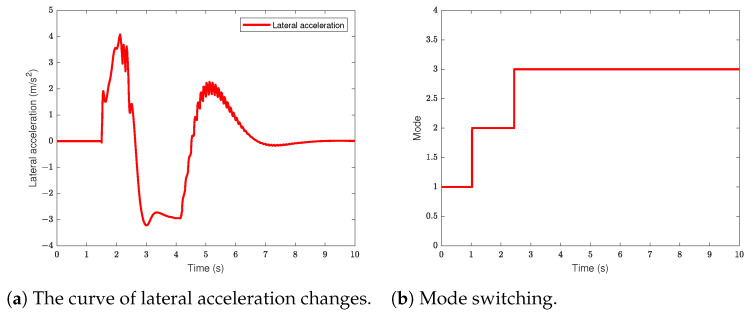
Scenarios for Adaptive Cruise Control.

In this simulation section, the vehicle travels at a constant speed of 120 km/h on a straight road. There is a stationary obstacle at a distance of 70 m directly ahead of the vehicle. When the vehicle detects the obstacle, it first applies emergency braking to avoid it, then steers to avoid the obstacle before returning to the set speed. The simulation results are shown in Figure 5 and Figure 6.

From Figure 5a, it can be observed that the vehicle begins steering avoidance when it is 20 m away from the obstacle vehicle. The planned trajectory effectively avoids the obstacle and guides the vehicle back to the reference trajectory. There is slight overshoot in the tracking process at 150 m, but it quickly tracks the reference trajectory. Figure 5b illustrates the variation of the vehicle’s yaw angle during the steering avoidance process. The yaw angle changes smoothly during the steering process with a small magnitude, indicating good stability of the vehicle during steering. Figure 5c shows the speed variation curve of the vehicle. The reference speed represents the speed of the static obstacle ahead. After steering to avoid the obstacle, the vehicle accelerates to the set speed. From the figure, it can be seen that the vehicle undergoes deceleration and then acceleration from 0 to 2.47 s, reaching the set speed at the 8 s. The speed change process is relatively gentle, indicating good comfort during deceleration and acceleration. Figure 5d depicts the acceleration variation of the vehicle during braking and steering. From the figure, it can be seen that the vehicle undergoes mild deceleration in the first 1.1 s, with a maximum deceleration of 3.26 m/$s^{2}$. When the relative distance between the two vehicles satisfies the emergency braking strategy, the vehicle begins emergency braking with a maximum deceleration of 5.14 m/$s^{2}$. During the braking process, when the relative distance between the two vehicles satisfies the steering avoidance strategy, the vehicle begins steering. At the same time, the vehicle starts accelerating at 2.47 s, eventually reaching the set speed. Additionally, Figure 5d also reflects the smooth transition between emergency control strategy and steering avoidance control strategy, ensuring passenger comfort.

Figure 6a shows the lateral acceleration variation curve of the vehicle during the steering avoidance. It can be observed that the lateral acceleration of the vehicle does not exceed 0.4 g during the steering avoidance, indicating that the tires consistently operate within the linear range, thus satisfying the small angle assumption of the vehicle dynamics model. Figure 6b represents the vehicle’s operating modes during the entire braking and steering obstacle avoidance process. The period before 1.1 s represents the vehicle decelerating, from 1.1 s to 2.47 s represents the vehicle in emergency braking mode, and after 2.47 s represents the vehicle steering to avoid the obstacle and accelerating to the set speed. Figure 6b reflects the effectiveness of mode switching and indicates the feasibility of the control strategy.

### 5.3. Following Condition

**Figure 7:**
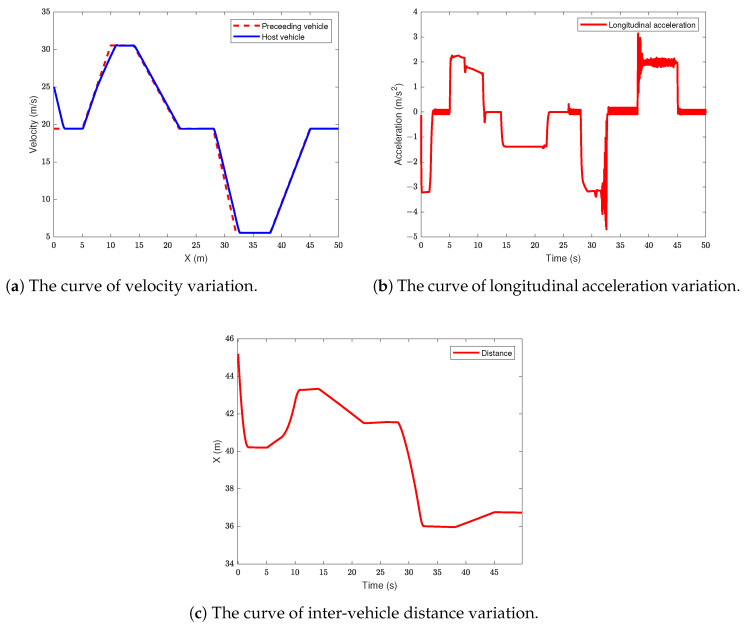
Scenarios for Adaptive Cruise Control.

In this section of the simulation, the initial velocity of the ego vehicle is set to 90 km/h, while the initial velocity of the lead vehicle is set to 70 km/h. The lead vehicle undergoes acceleration, deceleration, and steady-state driving within the interval of 0 to 50 s. During the following scenario, the ego vehicle uses the velocity of the lead vehicle as the reference speed, and the ACC system enters the distance control mode. Initially, the two vehicles are separated by a distance of 50 m. The simulation results are shown in Figure 7.

From Figure 7a,b, it can be observed that within the range of 0 to 50 s, the vehicle can effectively follow the preceding vehicle under various sub-conditions. Additionally, the speed variation curve responds rapidly. Although there was a slight deviation from the desired speed between 7 s to 10 s, the vehicle eventually manages to stably follow the preceding vehicle. During deceleration, the vehicle consistently maintains a safe distance from the preceding vehicle without collision. As shown in Figure 7c, within the initial 28 s, the vehicle maintains stable following with a maximum deceleration of 3.2 m/$s^{2}$ when the preceding vehicle suddenly decelerates to 20 km/h. The vehicle then initiates emergency braking with a maximum deceleration of 4.7 m/$s^{2}$, while the relative distance also decreases rapidly. When the preceding vehicle stabilizes, the vehicle’s acceleration remains steady.

We tested each of the three simulation scenarios five times under the same prediction and control time domain to verify the real-time performance of the MPC controller improved based on ADMM. This paper compares and analyzes the proposed algorithm with active set method (ASM) and interior point method (IPM). The processor parameters of the laptop used in the simulation are AMD Ryzen 7 5700U with Radeon Graphics 1.80 GHz. Table 2 shows the average computation time of the three methods throughout the entire obstacle avoidance process.

From above table, it can be seen that the improved controller has reduced computation time by 17.8% and 42.2% respectively under dual-shift-line scenarios, by 21.2% and 74.3% respectively under braking/steering scenarios, and by 18.3% and 30.7% respectively under the final scenario. The improved controller shows enhanced real-time performance compared to IPM and ASM. Improving controller real-time performance ensures rapid response to speed changes and enables swift navigation through obstacle trajectories, ensuring safety for autonomous vehicles during obstacle avoidance.

## 6. Conclusions

This paper addresses the situation where emergency braking alone cannot meet the obstacle avoidance requirements for autonomous vehicles. It proposes a control strategy combining adaptive cruise control with obstacle avoidance functions, introducing an emergency obstacle avoidance feature. The overall control mode is divided into following mode, emergency braking mode, and steering avoidance mode. In the emergency avoidance mode, nonlinear model prediction is used for local path planning based on the vehicle’s kinematic model. A quintic polynomial is employed for fitting. In terms of control strategy, the vehicle’s emergency steering distance is computed and combined with the obstacle avoidance function. This allows the vehicle to perform emergency avoidance while ensuring driving safety. Simulations are conducted under three different scenarios: dynamic obstacle avoidance with double lane changes, emergency braking and steering, and following scenarios. Results from various simulation scenarios indicate that the designed controller can quickly and stably track the preceding vehicle’s speed while maintaining a safe distance. After avoiding obstacles, the vehicle can return to the reference trajectory. During obstacle avoidance, the lateral angle changes smoothly with small fluctuations, ensuring vehicle stability. In emergency braking and steering avoidance scenarios, the lateral acceleration remains within 0.4 *g*, and the mode switches smoothly and rapidly. The proposed control strategy meets the requirements of responsiveness, stability, and comfort, allowing for obstacle avoidance through emergency braking or steering in critical situations.

## Figures and Tables

**Table 1:** Vehicle parameters.

Parameters	Value	Unit
Vehicle weight	1723	kg
Wheelbase	2.7	m
lateral moment of inertia	4331.6	kg · $m^{2}$
front and rear axle roll stiffness	2328/2653	N · m/rad
front and rear axle roll damping	47,298/37,311	N · m/rad
wheel lateral stiffness	61,900	N/rad
wheel rotational inertia	0.9	kg · $m^{2}$

**Table 2:** Average calculation time of the controller.

Scenarios	Average Computation Time for Scenario 1 (*s*)			Average Computation Time for Scenario 2 (*s*)			Average Computation Time for Scenario 3 (*s*)		
Method	ADMM-MPC	ASM	IPM	ADMM-MPC	ASM	IPM	ADMM-MPC	ASM	IPM
Test 1	0.0712	0.0854	0.1254	0.0517	0.0685	0.2124	0.3124	0.3795	0.4657
Test 2	0.0724	0.0920	0.1221	0.0514	0.0654	0.2024	0.3246	0.3469	0.4950
Test 3	0.0719	0.0831	0.1383	0.0602	0.0712	0.2314	0.3519	0.3912	0.4631
Test 4	0.0832	0.1055	0.1249	0.0531	0.0679	0.1987	0.2997	0.4006	0.4658
Test 5	0.0706	0.0847	0.1267	0.0529	0.0687	0.2013	0.3473	0.3570	0.4725

## Data Availability

Data is contained within the article.
